# Simulation of ultrafast electron diffraction intensity under coherent acoustic phonons

**DOI:** 10.1063/4.0000199

**Published:** 2023-11-13

**Authors:** Yongzhao Zhang, Jun Li, Wentao Wang, Huanfang Tian, Wenli Gao, Jianqi Li, Shuaishuai Sun, Huaixin Yang

**Affiliations:** 1 Beijing National Laboratory for Condensed Matter Physics, Institute of Physics, Chinese Academy of Sciences, Beijing 100190, People's Republic of China; 2 Institute of Quantum Materials and Physics, Henan Academy of Science, Zhengzhou 450046, China People's Republic of China; 3 School of Physical Sciences, University of Chinese Academy of Science, Beijing 100190, People's Republic of China; 4 School of Physics, Northwest University, Xi'an 710069, People's Republic of China; 5 Songshan Lake Materials Laboratory, Dongguan, Guangdong 523808, People's Republic of China; 6 Yangtze River Delta Physics Research Center Co., Ltd., Liyang, Jiangsu 213300, People's Republic of China

## Abstract

Ultrafast electron diffraction has been proven to be a powerful tool for the study of coherent acoustic phonons owing to its high sensitivity to crystal structures. However, this sensitivity leads to complicated behavior of the diffraction intensity, which complicates the analysis process of phonons, especially higher harmonics. Here, we theoretically analyze the effects of photoinduced coherent transverse and longitudinal acoustic phonons on electron diffraction to provide a guide for the exploitation and modulation of coherent phonons. The simulation of the electron diffraction was performed in 30-nm films with different optical penetration depths based on the atomic displacements obtained by solving the wave equation. The simulation results exhibit a complex relationship between the frequencies of the phonons and diffraction signals, which highly depends on the laser penetration depth, sample thickness, and temporal stress distribution. In addition, an intensity decomposition method is proposed to account for the in-phase oscillation and high harmonics caused by inhomogeneous excitation. These results can provide new perspectives and insights for a comprehensive and accurate understanding of the lattice response under coherent phonons.

## INTRODUCTION

The generation and propagation of coherent acoustic phonons can produce periodic modulation of the crystal structure, which exists as collective atomic motion, and induce phase transition.^1–4^ Upon femtosecond-laser excitation, coherent longitudinal acoustic (LA) waves usually appear in the material due to thermoelastic effects or deformation potential generated by the carriers in the sample.^5,6^ The excitation of the coherent transverse acoustic (TA) waves is noted when the instability of the crystal structure or disoriented lattice is considered.^7–10^ In addition, several works have investigated the mechanism of piezoelectrically induced coherent phonons.^11,12^

Laser-induced coherent phonons typically contain multiple frequencies. In thick films, where the optical penetration depth is considerably smaller than the sample thickness, a spatially non-uniform stress distribution in longitudinal direction is excited. Both even and odd high harmonics can be observed in the coherent oscillation, corresponding to the propagating strain waves bouncing back and forth in the film.^13,14^ When the optical penetration depth is much larger than the thickness, uniform stress is generated in the film, resulting in a standing wave with odd high harmonics only.^13,15^ The modulation effect of coherent acoustic phonons on the crystal structure makes them easily detectable by ultrafast electron diffraction (UED). However, common samples used in UED measurements have an optical penetration depth comparable to the film thickness,^16^ where the traveling and standing waves can coexist in the system, thereby complicating the analysis. In particular, LA and TA phonons can be simultaneously excited in a given system, generating complex multifrequency oscillation signals.^8,17^ Structural fluctuations induced by coherent phonons can be manifested in the diffraction intensity. A crucial yet rarely discussed issue is whether the experimentally observed oscillation frequencies in the diffraction can directly reflect the intrinsic phonon frequencies.^8,9,18^ Recently, the complex behavior of electron diffraction under high harmonics and lattice distortion in graphite nanofilms has been discussed in detail, raising the possibility of the selective excitation of higher harmonics.^19^ However, several issues are yet to be discussed in detail, such as the effect of higher harmonics in TA phonons on the diffraction and mechanisms of in-phase and out-of-phase oscillation of the Friedel pairs.^20^

In previous several UED works^8,9,17,20^ reporting diffraction intensity oscillation in the tens of GHz range, multiple-frequency is usually attributed to the coexistence of TA and LA phonons, and in-phase oscillation is considered to be caused by initial deviation parameter. Here, we propose that the inhomogeneous of laser excitation has a strong influence on the diffraction intensity (both oscillation phase and frequency). In this work, we theoretically analyzed the complex correspondence between the frequencies of laser-induced coherent acoustic phonons, including the TA and LA modes, and electron diffraction intensity in two hypothetical 30-nm films with laser penetration depths of 10 and 600 nm. In addition, several excitation profiles with the rise time of 0–60 ps were used to investigate their influence on the atomic motion to give a comprehensive understanding of diffraction signal, such as the phase shift of oscillations observed in previous works.^21–24^ Our results could give a new interpretation of the high harmonic diffraction signals, in-phase and out-of-phase oscillations, and provide further understanding of the uniformity of the laser distribution.

## EXCITATION CONDITIONS

**FIG. 1.**
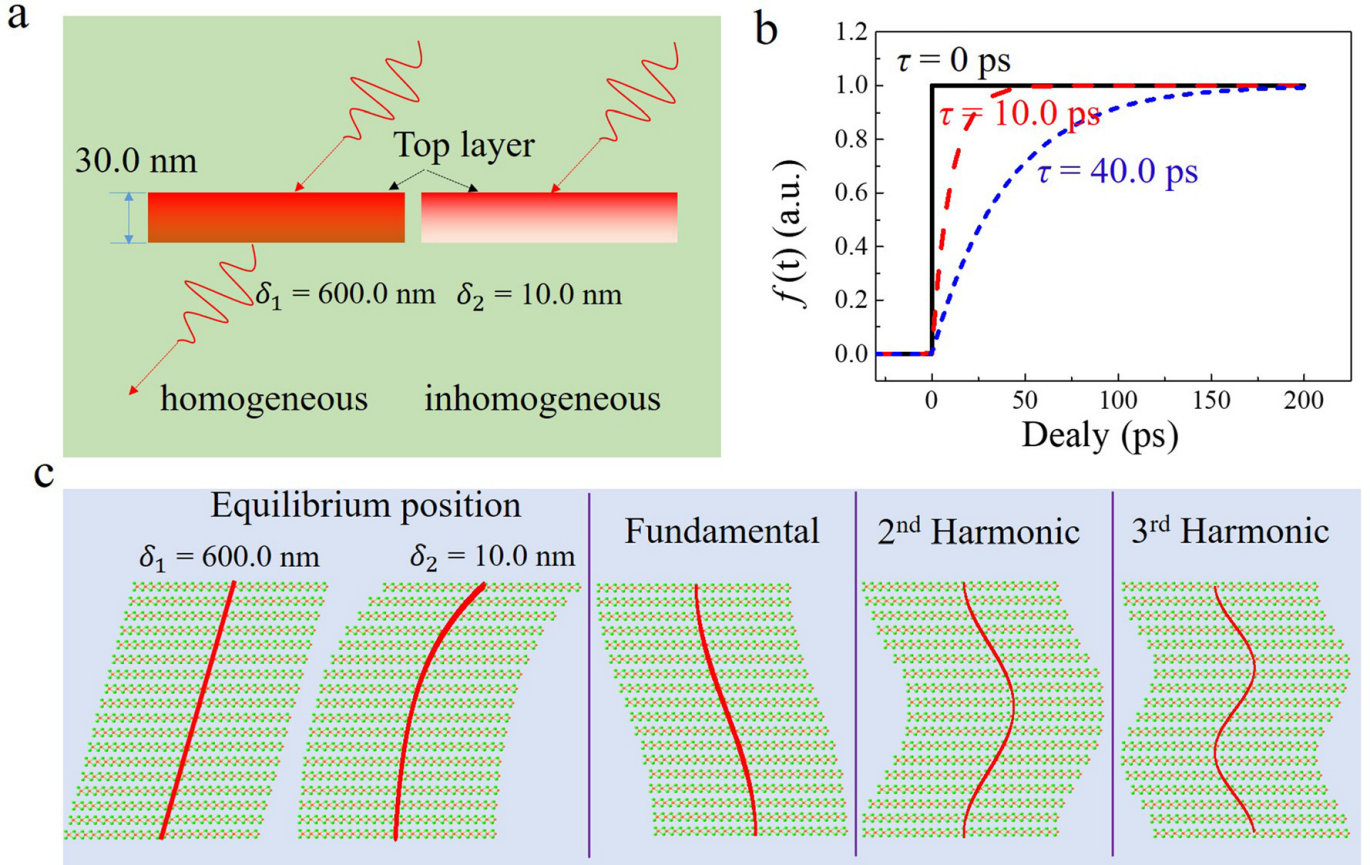
Atomic motion under coherent acoustic phonons. (a) Laser condition used in the calculation. (b) Temporal excitation profile of stress $\sigma_{\text{ext}}$ in the calculation. (c) Schematic of the atomic displacements in shear mode. The total oscillation is the superposition of the equilibrium position, fundamental, and higher harmonics.

We specified that the laser is first irradiated on the upper surface of the sample, and the film thickness was set to 30 nm. Considering the region of interest (center of the Gauss laser) and timescale (<10 ps), a uniform excitation profile in the lateral direction is assumed, and the diffusion of the carrier and heat along the lateral direction are ignored. However, the longitudinal excitation profile is distinct due to the difference in the optical penetration depth. The lattice evolution under homogeneous and inhomogeneous excitation is obtained by assuming different optical absorption coefficients $\alpha_{1}$ and $\alpha_{2}$. As shown in Fig. 1(a), the distribution of the laser energy in the film is approximately uniform when the penetration depth $\delta_{1}$ ( $1/\alpha_{1}$) is 600 nm. Meanwhile, when $\delta_{2}$ ( $1/\alpha_{2}$) is 10 nm, a significant inhomogeneity is created with the laser confined to a limited film depth. The carrier and heat diffusion along the longitudinal direction will smooth the inhomogeneity of laser deposited energy in different timescale. The carrier diffusion mainly occurs before the rapid relaxation through electron-phonon coupling (∼ps), then the heat diffusion dominates the thermalization within the sample, which may take several nanoseconds for a thickness of 30 nm, depending on thermal diffusivity.^25,26^ To simplify, we ignore the carrier and thermal diffusion along the longitudinal direction.

Compared with the LA breathing mode routinely excited in thin films caused by thermal-elastic stress, the photo-excitation of coherent TA shear modes requires sophisticated experimental configurations. There have been several articles reporting ultrafast coherent shear modes driven by different mechanism (transverse inhomogeneous excitation in Si;^15^ inverse piezoelectric effect in BiFeO_3_;^11^ structural instability in VTe_2_;^8^ miscut along a low symmetry face in TaAs;^27^ demagnetization in FePS_3_^28^). The common feature for them is the symmetry breaking in the surface plane. In order to make the excitation of TA phonons reasonable, we assume that the film has a monoclinic crystal structure with lattice constants a = 3.52Å, b = 3.52 Å, c = 7 Å, α = 90°, β = 90.3°, and γ = 90°. The structure may appear to shear along the b-direction under laser excitation due to the structural instability. In laser-induced breathing mode, the atomic displacements are typically proportional to the laser fluence. To more conveniently study the behavior of electron diffraction under different shear displacements, we assume that the amplitude of the shear mode and the laser are also positively proportional, just like the breathing mode. In this case, the amplitudes of both shear and breathing of the lattice can be wrote as βQ, where Q is the absorbed laser fluence, and β is the linear expansion/shear ratio with respect to the lattice constant induced by the unit absorbed laser energy. The stress induced by the laser excitation can be denoted by $\sigma_{\text{ext}}$ as a function of time *t* and film depth *z*.^29^ The temporal response of the atomic motion would exhibit sine-like (same as impulsive excitation^30^) or cosine-like (same as displacive excitation^31^) oscillation depending on the excitation mechanisms. In our calculation, the excitation temporal response used is step-function like, as shown in Fig. 1(b), which is described with exponential functions with different rise times τ, and indicates the absorption of laser energy by the sample is finally stored in the lattice. In this case, all atoms are driven to a new equilibrium position, corresponding to the cosine-like response. Furthermore, given the exponential decay of laser fluence inside the sample, the stress can be expressed as

$$\sigma_{\text{ext}}\left(z,t\right)=-C\beta \text{Q f}\left(t\right)g\left(z\right)=-C\beta Q\left(1-\text{exp}\left(-\frac{t}{\tau }\right)\right)\text{exp}(-\alpha z),$$ (1)

where C is the elastic modulus of the crystal along different directions, which is Young's modulus *E* and shear modulus *G* for the breathing and shear mode, respectively. The values of these parameters used are listed in Table I, which are set according to the typical values of two dimensional van der Waals materials.^32^

**TABLE I.** Parameters used in the calculation of atomic motion.

Definition	Symbol	Value
Elastic modulus	E, G	50, 22 GPa
Linear expansion/shear rate	β	2.5 × 10^−3^ (mJ/cm^2^)^−1^
Absorbed laser fluence	*Q*	2.0 mJ/cm^2^
Density	ρ	5.5 g/cm^3^
Velocity of strain wave	$v_{TA}$, $v_{LA}$	2.0, 3.0 km/s
Absorption coefficient	$\alpha_{1}$, $\alpha_{2}$	1.7 × 10^3^, 1.0 × 10^6^ cm^−1^

## CALCULATION OF THE ATOMIC DISPLACEMENTS

The generation and propagation of the coherent acoustic phonons can be described by the atomic displacement $\mu \left(z,t\right)$ calculated from the wave equation in continuous medium.^5^ When the laser beam diameter is considerably larger than the laser penetration depth, it can be assumed that only the coherent phonons propagating along the out-of-plane direction are triggered in the region far from the sample edge. Thus, the wave equation can be directly expressed in one-dimensional form, as follows:

$$\frac{\partial^{2}\mu }{\partial t^{2}}=v^{2}\frac{\partial^{2}\mu }{\partial z^{2}}+\frac{\lambda }{\rho }\frac{\partial \mu }{\partial t}+\frac{\partial \sigma_{\text{ext}}}{\rho \partial z},$$ (2)

where λ is the damping factor that determines the lifetime of the coherent phonons inside the film, which is set as zero for explicitly. The velocity of the out-of-plane TA and LA wave is set as $v_{TA}=2.0$ and $v_{LA}=3.0\;\mathrm{km}/s$, respectively. All the parameters used for our numerical calculation are listed in Table I. For a freestanding film, the free boundary conditions is

$$\left.\frac{\partial \mu }{\partial z}\right|\left(z=0,t\right)=\left.\frac{\partial \mu }{\partial z}\right|\left(z=d,t\right)=0,$$ (3)

where *d* is the thickness of the film. Steady-state solutions to the wave Eq. (2) under free boundary conditions and the initial condition $\left.\frac{\partial \mu }{\partial t}\right|\left(z,0\right)=0$ can be described as a superposition of standing waves,

$${\mu \left(z,t\right)|}_{f_{j\neq 0}}=A_{j}\cdot \text{cos}\left(\frac{j\pi }{d}z\right)\text{sin}(2\pi f_{j}\cdot t+\varphi_{j}).$$ (4)

The value of amplitude $A_{j}$ depends on the temporal and spatial distribution of stress $\sigma_{\text{ext}}$, which will be discussed in following text. The frequency of the jth mode has the form $f_{j\neq 0}=jv/2d$. In a cosine-like response, the phase $\varphi_{j}$ has the value of π/2. Here, we defined the new atomic equilibrium position after laser excitation as ${\mu \left(z,t\right)|}_{f_{j=0}}$, in which $f_{j=0}=0$. The actual displacement of the atoms $\mu \left(z,t\right)$ at depth *z* is the sum of ${\mu \left(z,t\right)|}_{f_{j\neq 0}}$ and ${\mu \left(z,t\right)|}_{f_{j=0}}$ with the form

$$\mu \left(z,t\right){|}_{f_{j=0}}=\mu \left(z,0\right)-\sum_{j}A_{j}\cdot \text{cos}\left(\frac{j\pi }{d}z\right),$$ (5)

where $\mu \left(z,0\right)$ is the initial displacement of each atoms at depth *z*. In a perfect crystal, $\mu \left(z,0\right)=0,$ whereas in an actual crystal, $\mu \left(z,0\right)$ has nonzero values for a disordered structure. Substituting the stress $\sigma_{\text{ext}}$ [Eq. (1)] into the wave Eq. (2), the atomic displacements in the shear or breathing mode can be numerical solved with the initial condition $\mu \left(z,0\right)=\left.\frac{\partial \mu }{\partial t}\right|\left(z,0\right)=0$ and the free boundary conditions (3). Figure 1(c) shows the calculated equilibrium position ${\mu \left(z,t\right)|}_{f_{j=0}}$ and oscillation mode $A_{j}\cdot \text{cos}\left(\frac{j\pi }{d}z\right)$ in shear mode (*j* = 1, 2, and 3, corresponding to the fundamental, second harmonic, and third harmonic mode, respectively).

**FIG. 2.**
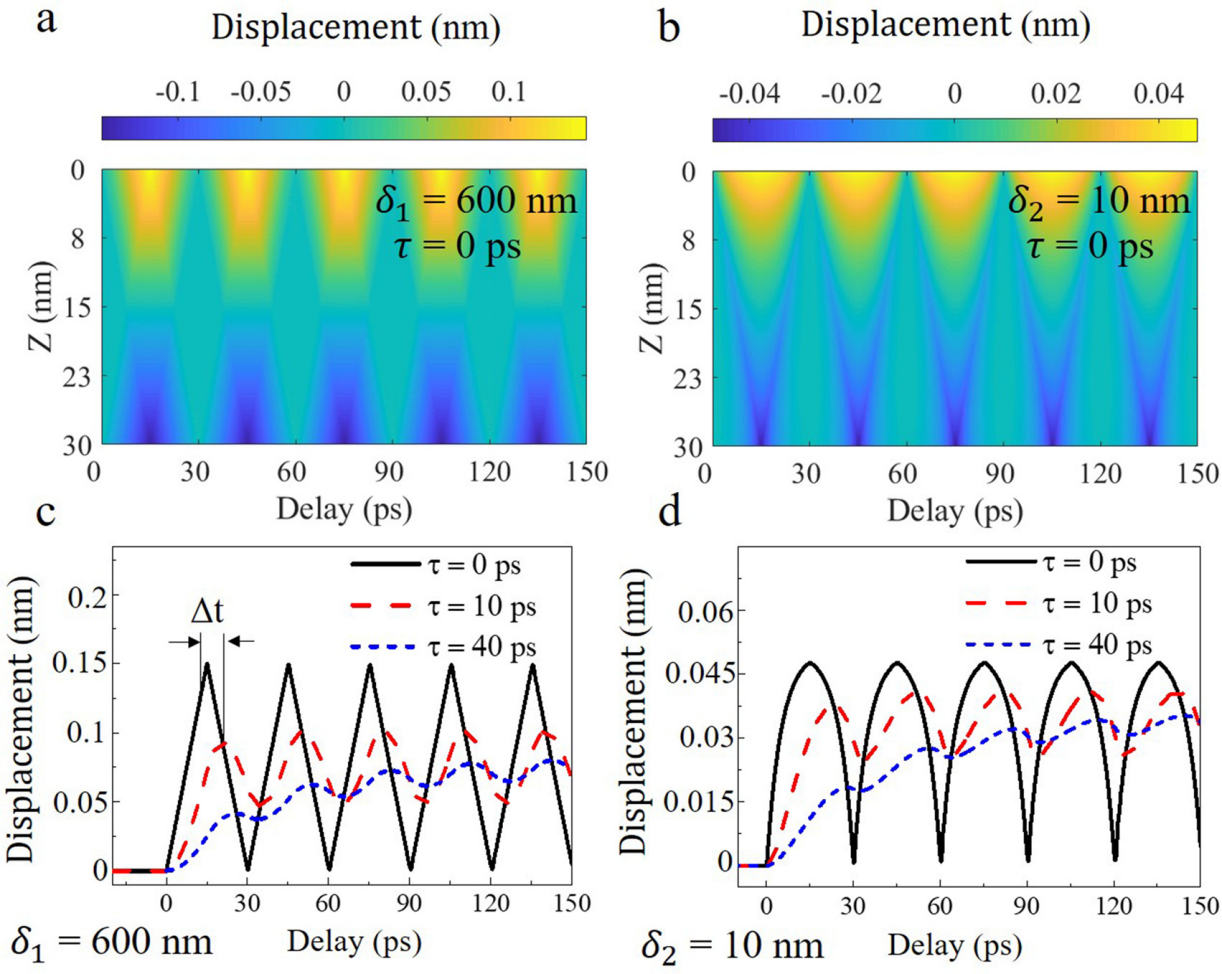
Atomic motions with different rise times *τ* of the stress $\sigma_{\text{ext}}$. (a, b) Dynamic simulation of depth-dependent atomic displacement in the two films with optical penetration depth of 10 and 600 nm, respectively. The rise time τ used is 0 ps. (c) Atomic motions at the top surface with the rise time *τ* of 0, 10, and 40 ps in the film with the optical penetration depth $\delta_{1}=$ 600 nm. (d) Corresponding information of the film with the optical penetration depth $\delta_{2}=$ 10 nm.

Dynamic simulation of depth-dependent atomic displacement in the two films with optical penetration depth of 10 and 600 nm is shown in Figs. 2(a) and 2(b), respectively (The rise time *τ* = 0 ps). The homogeneous absorption of laser leads to the excitation of a typical standing wave. However, for inhomogeneous absorption, in addition to the standing wave, a traveling wave propagating along the surface normal becomes evident. Figure 2(c) plots the calculated shear displacements at the top layer of the film (z = 0) with the optical penetration depth $\delta_{1}=$ 600 nm, showing an oscillation period *T* = 30 ps. The position of the first peak and amplitude of the oscillations under different rise times exhibits distinct features. The longer rise time *τ* could attenuate the amplitude and push oscillation toward a positive direction. We used Δt to denote the shift of the first peak position relative to the position, where delay = *T*/2 = 15 ps. The phase shift 2πΔt/T will increase with the rise time *τ*, but eventually converge to π/2 which can be analyzed by Green function. The corresponding information on the film with the optical penetration depth $\delta_{2}=$ 10 nm is shown in Fig. 2(d), in which the phase shift and amplitude attenuation exhibit similar behaviors as in the homogeneous condition.

**FIG. 3.**
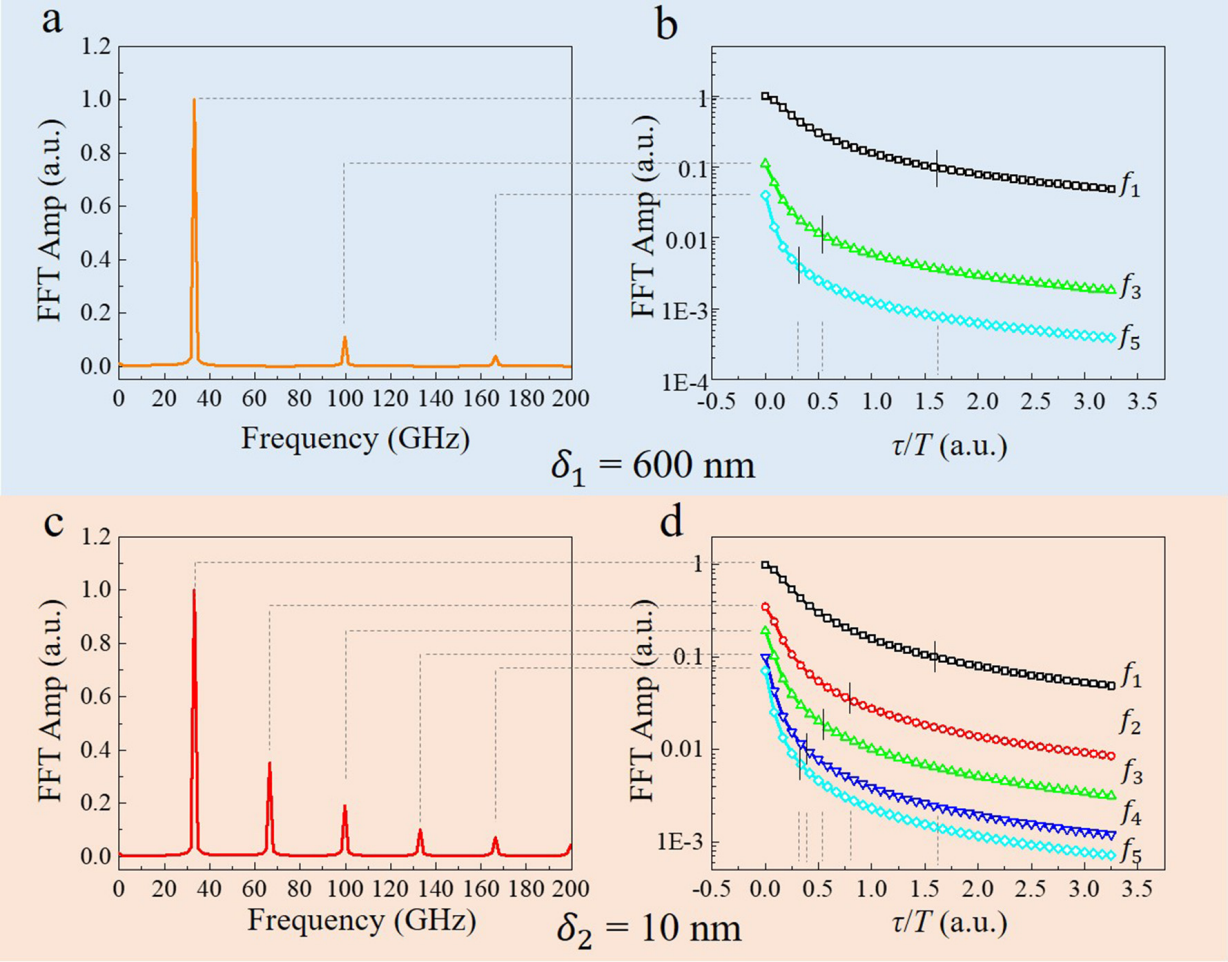
Amplitude suppression effect related to the rise time *τ* of the stress $\sigma_{\text{ext}}$. (a) FFT of the atomic displacements at the top surface under homogeneous excitation with *τ/T* = 0. (b) Amplitude of the frequency in (a) against the rise time *τ/T*. (c, d) Corresponding information under inhomogeneous excitation.

To quantify the effect of the rise time *τ* on the oscillation amplitude with the specific frequency, fast Fourier transform (FFT) was conducted on the oscillation profile [black curves in Figs. 2(c) and 2(d)] with the rise time *τ* = 0 ps, as shown in Fig. 3(a) and 3(c). By performing FFT of atomic motions at the top surface calculated with different rise time *τ,* the amplitude of each frequency against *τ/T* can be extracted as shown in Fig. 3(b) and 3(d), in which the positions where the amplitude drops by one order of magnitude are marked with the vertical black lines. The results shown in Fig. 3 indicate the suppressed oscillation in both homogeneous and inhomogeneous excitation as the rise time increases. In addition, the higher harmonics are more sensitive to the rise time. It is worth noting that there is only odd harmonics under homogeneous excitation, where the longitudinal excitation profile (and also the boundary condition) has central inversion symmetry about the center of the sample; however, the even harmonics appears under inhomogeneous excitation where the inversion symmetry is broken.

## SIMULATION AND DECOMPOSITION OF THE DIFFRACTION INTENSITY

**FIG. 4.**
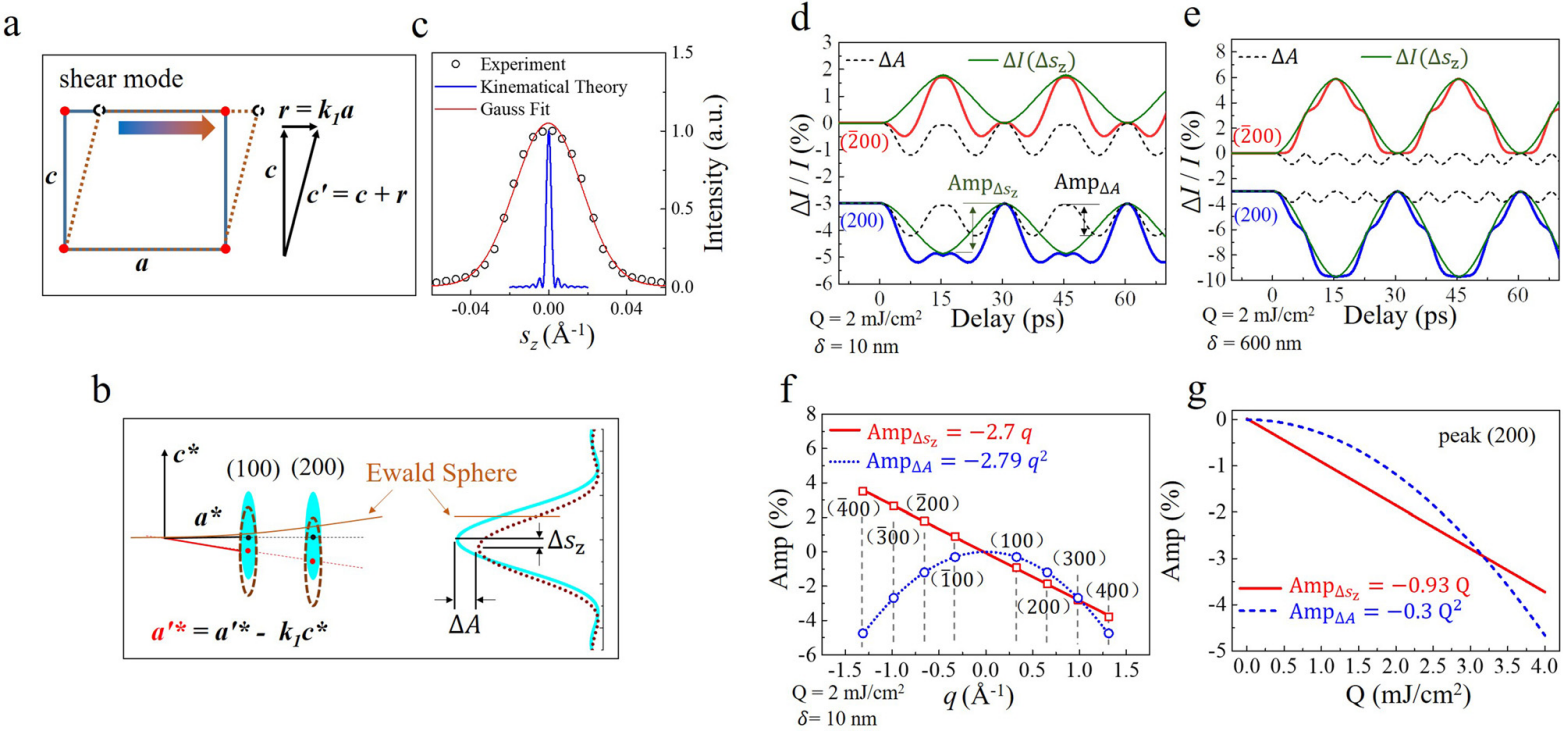
Decomposition of the diffraction intensity. (a) Schematic of the shear mode. (b) Influence of the shear mode on the reciprocal rod. (c) Distinction between the experimentally detected and theoretically calculated intensity distribution against the deviation parameter $s_{z}$ in the 27-nm MoTe_2_ film. The red line shows the Gaussian fit of the reciprocal rod. (d) Intensity simulation and decomposition of the Friedel pairs (200) and $\left(\bar{2}00\right)$ after laser excitation with the fluence of 2 mJ/cm^2^ and penetration depth of 10 nm. Total intensity change $\Delta I/I\approx \Delta A+\Delta I\left(\Delta s_{z}\right)$. The red and blue curves represent the intensity change of the Bragg peaks (200) and $\left(\bar{2}00\right)$, respectively. Green solid line: intensity change $\Delta I\left(\Delta s_{z}\right)$ caused by the shift $\Delta s_{z}$ of the reciprocal rod. Black dash line: amplitude change ΔA of the reciprocal rod. (e) Corresponding information of the film with the optical penetration depth δ= 600 nm. (f) The respective oscillation amplitude ${\mathrm{Amp}}_{\Delta s_{z}}$ and ${\mathrm{Amp}}_{\Delta A}$ of $\Delta I\left(\Delta s_{z}\right)$ and ΔA in the Bragg peaks with different scattering vectors. (g) The values of ${\mathrm{Amp}}_{\Delta s_{z}}$ and ${\mathrm{Amp}}_{\Delta A}$ under different laser fluences.

To investigate the effect of the coherent phonon modes on the diffraction, kinematical diffraction simulations were performed in the proposed crystal ( a = 3.52 Å, b = 3.52 Å, c = 7 Å, α = 90°, β = 90.3°, γ = 90°). The sample surface is parallel to the plane where lattice vectors a and b lie in, similar to the layered films obtained by mechanical exfoliation. In the calculation of the shear mode, the direction of the atomic displacement on the top surface is set along the a direction [Fig. 4(a)]. For the breathing mode, the displacement is along the $c^{*}$ direction. For the diffraction simulation and analyses in this and next section, the rise time τ in stress $\sigma_{\text{ext}}$ and damping factor *λ* are set as zero for simplicity. Subsequently, Eqs. (1) and (2) can be totally determined, and the atomic displacements $\mu \left(z,t\right)$ can be numerically solved, which are used for the simulation of the diffraction in the following text.

In the crystal containing $N_{x}$, $N_{y}$, and $N_{z}$ unit cells along the three fundamental vectors a, b, and c, the reciprocal rod [right of Fig. 4(b)] of Bragg peak with the reciprocal vector of G can be obtained by introducing the calculated atomic displacements $\mu \left(z,t\right)$ into the kinematical diffraction equation^33^

$$I_{\text{kin}}\left(s,t\right)\propto {\left|A\right|}^{2}=\frac{{\text{sin}}^{2}(\pi N_{x}G\cdot a)}{{\text{sin}}^{2}(\pi G\cdot a)}\frac{{\text{sin}}^{2}(\pi N_{y}G\cdot b)}{{\text{sin}}^{2}(\pi G\cdot b)}{\left|\sum_{n_{z}=1}^{N_{z}}F_{n_{z}}\text{exp}[2\pi iG\cdot {(r}_{n}+\mu \left(z,t\right))]\right|}^{2},$$ (6)

where $r_{n}$ is the lattice position with $r_{n}=n_{x}a\;{+n}_{y}b\;{+n}_{z}c$. $F_{n_{z}}$ is the structure factor of the unit cell at $n_{z}$-th layer. For the diffraction simulation, we propose a decomposition method to offer a new perspective on the diffraction intensity in ultrafast experiments. As shown in Figs. 4(a) and 4(b), when lattice vectors c changes to $c^{\prime }=c+r$ with the shear displacement $r=k_{1}a$, reciprocal vector $a^{\prime *}=b\times (c+r)/V=a^{*}-k_{1}c^{*}$. Similarly, if $r=k_{2}b$, reciprocal vector $b^{\prime *}=b^{*}-k_{2}c^{*}$. As a result, all Bragg peaks (hkl) with h ≠ 0 will undergo a shift $\Delta s_{z}$ = ${-hk}_{1}c^{*}$ along the $c^{*}$ direction in reciprocal space, resulting in the intensity redistribution $I_{\text{kin}}^{\prime }\left(s_{z}\right)=I_{\text{kin}}\left(s_{z}-\Delta s_{z}\right)$ and the relative intensity change ${\Delta I\left({\Delta s}_{z}\right)|}_{s_{z}}=[I_{\text{kin}}^{\prime }(s_{z})-I_{\text{kin}}\left(s_{z}\right)]/I_{\text{kin}}\left(s_{z}\right)$, where $s_{z}$ is deviation parameter along the $c^{*}$ direction. Due to the different atomic displacements of each layer, $\Delta s_{z}$ should have the form $\Delta s_{z}\propto -h\bar{k_{1}}c^{*}=-hc^{*}\sum_{n_{z}=1}^{N_{z}}\left(k_{1,n_{z}}/N_{z}\right)$ for total crystal. In the kinematical diffraction theory [Eq. (6)], the diffraction intensity is the coherent superposition of each lattice layer. Meanwhile, the different vibration amplitudes of each layer reduce this coherence and introduce decrease in diffraction amplitude for all peaks (hkl) with h ≠ 0 (in the case $r=k_{1}a$). We use $\Delta A=\left[I_{\text{kin}}^{\prime }\left({\Delta s}_{z}\right)-I_{\text{kin}}(0)\right]/I_{\text{kin}}(0)$ to describe the amplitude change of the reciprocal rod.

In the actual experiments, the flatness of the sample surface dominates the width of reciprocal rod,^9,20^ which can be one order of magnitude larger than the theoretical value determined by Eq. (6). We have experimentally measured the reciprocal rod length of 2H-MoTe_2_ thin films with a thickness of 27 nm and selected area of 5 *μ*m as shown in Fig. 4(c). The samples used for the measurement were obtained by mechanical exfoliation from a bulk crystal, and the thicknesses were determined by electron energy loss spectroscopy. The reciprocal rod was performed through the (110) peak with the electron beam incident along the [001] zone axis of the crystal. We use the Gaussian function to fit the experimentally measured results and substitute back into the simulation, which has the following form:

$$I_{\text{exp}}(s_{z})=\text{exp}\left(-\frac{2{s_{z}}^{2}}{\sigma^{2}}\right),$$ (7)

where σ = 0.035 Å^−1^. The intensity change ${\Delta I\left({\Delta s}_{z}\right)|}_{s_{z}}$ induced by ${\Delta s}_{z}$ should be calculated as ${\Delta I\left({\Delta s}_{z}\right)|}_{s_{z}}=[I_{\text{exp}}(s_{z}-\Delta s_{z})-I_{\text{exp}}\left(s_{z}\right)]/I_{\text{exp}}\left(s_{z}\right)$, while the amplitude change ΔA still has the form $\Delta A=\left[I_{\text{kin}}^{\prime }\left({\Delta s}_{z}\right)-I_{\text{kin}}(0)\right]/I_{\text{kin}}(0)$. The total intensity change at a specific deviation parameter $s_{z}$ can be decomposed as

$$\frac{\Delta I}{I}=\frac{\left(1+\Delta A)\cdot I_{\text{exp}}(s_{z}-\Delta s_{z}\right)}{I_{\text{exp}}\left(s_{z}\right)}-1=\Delta A[1+\Delta I\left(\Delta s_{z}\right)]+\Delta I\left(\Delta s_{z}\right)\approx \Delta A+\Delta I\left(\Delta s_{z}\right).$$ (8)

Under inhomogeneous excitation, the simulated temporal intensity evolutions of the Bragg peak (200) and $\left(\bar{2}00\right)$ with the same deviation parameter $s_{z}=0.01$Å^−1^ are indicated by the red and blue curves in Fig. 4(d), respectively. The corresponding intensity decomposition is shown by black dashed line ( $\Delta I\left(\Delta s_{z}\right)$) and green solid line ( ΔA). $\Delta I\left(\Delta s_{z}\right)$ mainly shows fundamental mode features (fundamental with *j* = 1 is dominant, with the oscillation period *T* = 30 ps), whereas high harmonic features are dominant in ΔA (second harmonic with *j* = 2 is dominant, with period of *T*/2). Another key point is that the oscillation of $\Delta I\left(\Delta s_{z}\right)$ in (200) and $\left(\bar{2}00\right)$ exhibits an out-of-phase feature [ ${\text{phase}}_{\left(200\right)}=\pi$, ${\text{phase}}_{\left(\bar{2}00\right)}=0$] whereas ΔA shows an in-phase oscillation (both phases are π) in the Friedel pairs. Typically, the Friedel pairs have the same diffraction intensity at the same initial deviation parameter ( $s_{0,G}=s_{0,-G}$), which is determined by the central symmetry of the structural factors. However, the $\Delta I\left(\Delta s_{z}\right)$ and ΔA would cause the unequal change of the diffraction in Friedel pairs **G** and –**G**. Oscillations caused by ΔA within a Friedel pairs are always in-phase, while whether oscillations caused by $\Delta I\left(\Delta s_{z}\right)$ within a Friedel pairs are out-of-phase or in-phase sensitively depends on the initial deviation parameter, which is related to the tilt angle. Corresponding information of the film with the optical penetration depth δ= 600 nm is shown in Fig. 4(e). In homogeneous excitation, intensity change $\Delta I\left(\Delta s_{z}\right)$ is dominant. In addition, $\Delta I\left(\Delta A\right)$ mainly shows the oscillation with period of *T*/4 and a very small amplitude.

In order to provide the dependence of $\Delta I\left(\Delta s_{z}\right)$ and $\Delta I\left(\Delta A\right)$ on scattering vector q, the intensity oscillation of peak (h00) (h = ±1, h = ±2, and h = ±3) at the same deviation parameter $s_{z}=0.01$Å^−1^ is calculated. We denote the oscillation amplitude of ΔA and $\Delta I\left(\Delta s_{z}\right)$ by ${\mathrm{Amp}}_{\Delta A}$ and ${\mathrm{Amp}}_{\Delta s_{z}}$, repectively, and specify Amp <0 when phase =π, Amp >0 when phase =0, by which the phase distribution can be directly observed from the sign of the amplitude in Figs. 4(f) and 4(g). The ${\mathrm{Amp}}_{\Delta s_{z}}$ and ${\mathrm{Amp}}_{\Delta A}$ are plotted against the scattering vector *q* in Fig. 4(f). For peak (h00), the shift distance $\Delta s_{z}\propto -h\bar{k_{1}}c^{*}$ is proportional to h. In addition, the intensity change can be approximately considered as linear with $\Delta s_{z}$, considering the shape of the reciprocal rod [Eq. (6) and Fig. 4(c)]. Thus, the ${\mathrm{Amp}}_{\Delta s_{z}}$ satisfies a linear relationship with the scattering vector *q*. While the ${\mathrm{Amp}}_{\Delta A}$ satisfies the linear relationship with $q^{2}$, which can be attributed to the disorder of the entire crystal. When treating the film as a supercell, ΔA can be analogous to the Debye–Waller effect, which shows a linear relationship with $q^{2}$, suggesting it is a collective intensity decrease due to the overall structural disorder in the sample. The opposite signs of ${\mathrm{Amp}}_{\Delta s_{z}}$ in (h00) and $\left(\bar{h}00\right)$ indicate the out-of-phase feature [ ${\mathrm{Phase}}_{\left(h00\right)}=\pi ,\;{\mathrm{Phase}}_{\left(\bar{h}00\right)}=0$], whereas the oscillation of ΔA shows a consistent phase of π in all eight Bragg peaks. In addition, we calculated the diffraction intensity of peak (200) at different laser fluence (Q) and performed a similar intensity decomposition [Fig. 4(g)]. As mentioned above, $\Delta s_{z}\propto -h\bar{k_{1}}c^{*}$. Given the linear shear rate *β*, the mean expansion rate $\bar{k_{1}}\propto Q$. Thus, ${\mathrm{Amp}}_{\Delta s_{z}}$ satisfies an approximately linear relationship with the energy density Q. However, ${\mathrm{Amp}}_{\Delta A}$ depends on the coherence of each lattice layer, which is dramatically affected by the lattice disorder. We used the variance ( S) of lattice constant $c^{\prime }$ under the modulation of coherent phonons to describe the disorder, expressed as

$$S=\sum_{n_{z}}{\left(c_{n_{z}}^{\prime }-\bar{c^{\prime }}\right)}^{2}\propto \sum_{n_{z}}{\left(k_{{1,\;n}_{z}}-\bar{k_{1}}\right)}^{2}\propto {k_{{1,\;n}_{z}}}^{2}\propto Q^{2},$$ (9)

which is based on the relation $c_{n_{z}}^{\prime }=c-k_{1,n_{z}}a$ and $\bar{c^{\prime }}=\sum_{n_{z}}\left(c_{n_{z}}^{\prime }/N_{z}\right)$. Equation (9) achieved $S\propto Q^{2}$. The linear relationship between ${\mathrm{Amp}}_{\Delta A}$ and $Q^{2}$ shown in Fig. 4(g) indicates the amplitude of the reciprocal rod is proportional to the variance S.

**FIG. 5.**
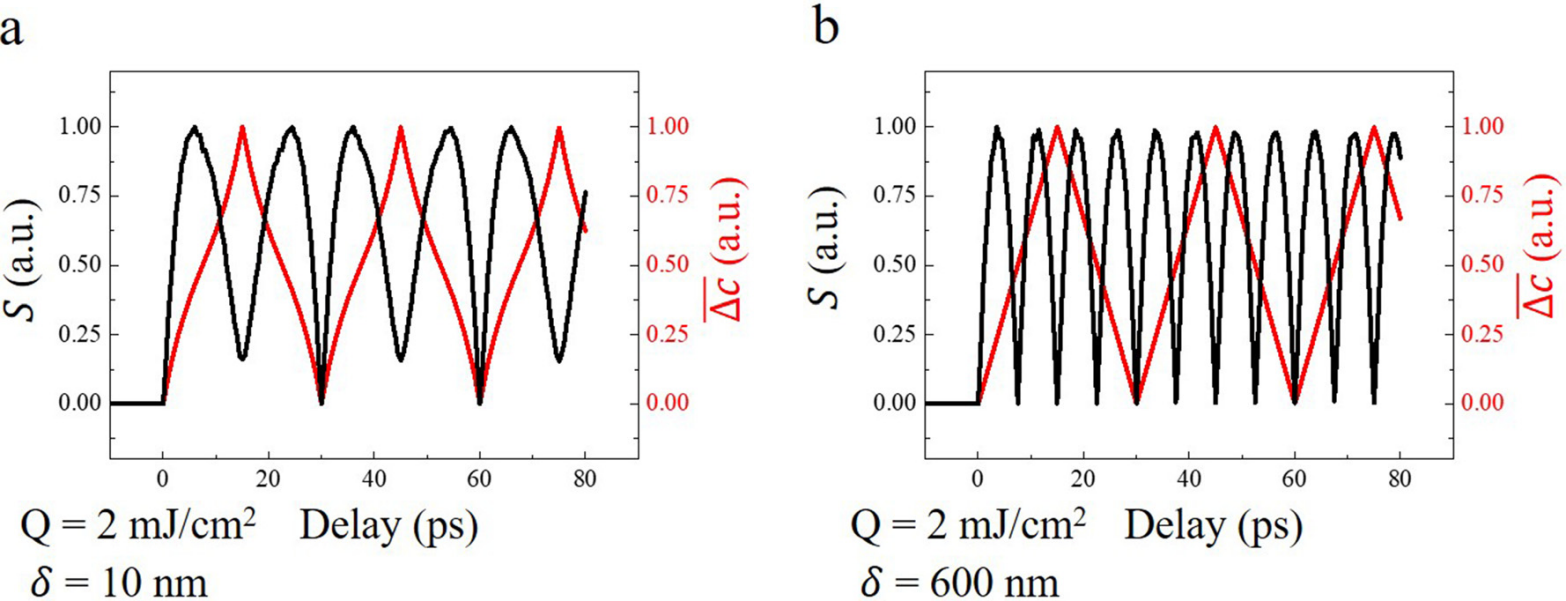
Temporal evolution of mean change [ $\bar{{\Delta c}^{\prime }}=\sum_{n_{z}}(c_{n_{z}}^{\prime }-\bar{c^{\prime }})/N_{z}$] and the variance $\left[S=\sum_{n_{z}}{\left(c_{n_{z}}^{\prime }-\bar{c^{\prime }}\right)}^{2}\right]$ of lattice constant with the modulation of shear mode. (a) and (b) show the corresponding results in inhomogeneous ( δ = 10 nm) and homogeneous excitation ( δ = 600 nm), respectively.

We use the mean change of lattice constant $\bar{{\Delta c}^{\prime }}$ ( $\bar{{\Delta c}^{\prime }}=\sum_{n_{z}}(c_{n_{z}}^{\prime }-\bar{c^{\prime }})/N_{z}$) to denote the mean lattice expansion or shearing. Figure 5 plots the temporal evolution of $\bar{{\Delta c}^{\prime }}$ and S with the modulation of shear mode in the crystal. It is clear that the oscillation of $\bar{{\Delta c}^{\prime }}$ shows an period of 30 ps, while S show high harmonic features, which is consistent with the results shown in Figs. 4(d) and 4(e). These results also support the conclusion that the intensity change $\Delta I\left(\Delta s_{z}\right)$ is mainly caused by the mean change of lattice, while $\Delta I\left(\Delta A\right)$ is stemming from the lattice disorder.

## FREQUENCY ANALYSIS

**FIG. 6.**
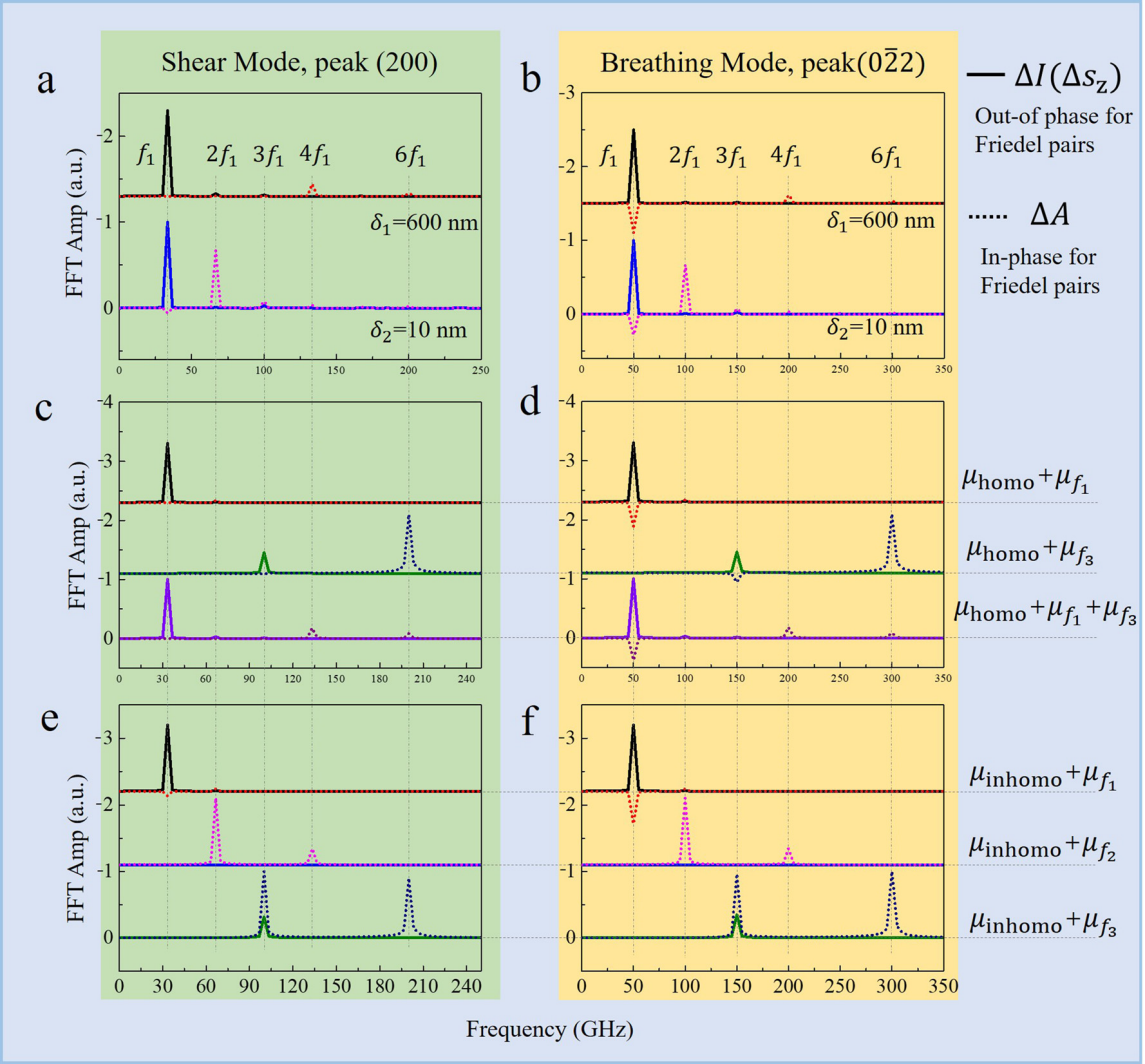
Frequency domain information of the diffraction intensity oscillation. (a) and (b) FFT of the (200) and $\left(0\bar{2}2\right)$ decomposed diffraction intensity in shear and breathing modes under homogeneous and inhomogeneous excitation. (c) and (d) FFT of the (200) and $\left(0\bar{2}2\right)$ diffraction intensity in shear and breathing modes with the specific phonon frequency based on the atomic equilibrium position in the homogeneous condition. (e) and (f) FFT of the (200) and $\left(0\bar{2}2\right)$ diffraction intensity in shear and breathing modes with the specific phonon frequency based on the equilibrium position in inhomogeneous condition.

After decomposing the intensity, the frequency distribution of the diffraction intensity and its relationship to the coherent phonon mode can be clarified. The shear modes can induce the intensity change of the in-plane peaks, whereas the breathing modes exert influence on the out-of-plane peaks. Given that the strain wave propagates along the reciprocal vector $c^{*}$, the FFT of the diffraction intensity of the peak (200) and $\left(0\bar{2}2\right)$ is performed to analyze the influence of the TA phonons (shear mode) and LA phonons (breathing mode), respectively. The FFT results of peak (200) under shear mode induced by homogeneous ( $\delta_{1}=$ 600 nm) and inhomogeneous ( $\delta_{2}=$ 10 nm) excitation is shown in Fig. 6(a), where the upper and lower curves are obtained based on the temporal oscillation data shown in Figs. 4(f) and 4(d), respectively. Figure 6(b) shows the corresponding information of peak $\left(0\bar{2}2\right)$ under the breathing mode. It should be noted that, in order to view peak $\left(0\bar{2}2\right)$, the crystal should be tilted to [011] zone axis. In the actual experiment, we used the sine function $y=A_{i}\text{sin}(2\pi f_{i}t+\varphi_{i})$ to describe the oscillation with the specific frequency $f_{i}$. All the oscillations shown in Fig. 6 have a phase of 0 or π due to the used step-function-like temporal excitation profile ( $\sigma_{\text{ext}}$ with τ=0). We specify the amplitude of FFT as >0 when $\varphi_{i}=\pi$, while <0 when $\varphi_{i}=0$, resulting in the negative values shown in Fig. 6.

Figures 6(a) and 6(b) indicate that the homogeneous excitation mainly causes the shift of the reciprocal rod, which is manifested by the intensity oscillations $\Delta I\left(\Delta s_{z}\right)$ with frequency $f_{1}$. Meanwhile, the oscillation of ΔA at the frequency of ${4f}_{1}$ indicates slight changes in the amplitude of the reciprocal rod. In contrast, inhomogeneous excitation causes more drastic reductions in the intensity of the reciprocal rod, resulting in a strong oscillation of ΔA with frequency of ${2f}_{1}$. These findings apply to both the breathing and shear modes; however, the behavior in the breathing mode is more complex. As shown in Fig. 6(b), the breathing mode causes additional oscillation of ΔA with frequency $f_{1}$, which is not related to the uniformity of the optical excitation.

The frequency of atomic displacements under coherent phonons contains multiple components, mainly includes the fundamental ( $f_{1}$), second harmonic ( $f_{2}$), and third harmonic ( $f_{3}$) components. To analyze the origin of the various frequencies of the diffraction in Figs. 6(a) and 6(b), atomic displacements under each frequency component based on the equilibrium position are introduced into the diffraction simulation. The results are shown in Figs. 6(c)–6(f), in which $\mu_{\;\text{homo}}$ and $\mu_{\text{inhomo}}$ denote the atomic equilibrium position after laser excitation. While, $\mu_{f_{i}}$ represents the atomic displacements induced by the vibration with frequency $f_{i}$. Corresponding schematic can be seen in Fig. 1(c).

For the shear mode under homogeneous excitation [Fig. 6(c)], the fundamental mode (frequency: $f_{1}$) generates the oscillation of diffraction intensity $\Delta I\left(\Delta s_{z}\right)$ with the same frequency $f_{1}$, which behaves as an out-of-phase oscillation in the Friedel pairs. The third harmonic mode produces the oscillation of ΔA and $\Delta I\left(\Delta s_{z}\right)$ with frequencies of ${6f}_{1}$ and ${3f}_{1}$, respectively. Notably, the simultaneous presence of the fundamental and third harmonic modes exhibit a weak ${4f}_{1}$ ( ΔA) signal in the diffraction, indicating that the influence of each mode is not completely independent. Thus, the oscillation of ΔA exhibits ${4f}_{1}$ signals for both the shear and breathing modes in the film with the penetration depth of 600 nm. These conclusions remain valid for the breathing mode under homogeneous excitation [Fig. 6(d)], except the influence of the fundamental mode with the atomic displacements of $\mu_{\text{homo}}+\mu_{f_{1}}$, which causes additional oscillations of ΔA with frequency of $f_{1}$.

The comparison of Figs. 6(c) and 6(e) [or Figs. 6(d) and 6(f)] indicates that, for the inhomogeneous excitation, the oscillation of ΔA shows obvious frequency ${i\cdot f}_{1}$ under the *i*-th order harmonic (*i* > 1), which is absent under the homogeneous condition. In addition, the amplitude of the oscillation with frequency $2{i\cdot f}_{1}$ exhibits obvious attenuation, which is attributed to the asymmetric distribution of the atomic equilibrium position with respect to the center of the film. Diffraction signals in inhomogeneous excitation exhibit a ${2f}_{1}$ oscillation not only because of the displacement $\mu_{f_{2}}$ of atoms at the second harmonic, but also by the equilibrium position of the atoms $\mu_{\text{inhomo}}$. The equilibrium position of atoms in the oscillation has great effect on diffraction, that is the reason why $\mu_{\text{homo}}$+ $\mu_{f_{3}}$ and $\mu_{\text{inhomo}}$+ $\mu_{f_{3}}$ show significant difference in diffraction [Figs. 6(c) and 6(e), or Figs. 6(d) and 6(f)], and why the second harmonics manifested in the diffraction intensity oscillation is much stronger than the true phonon oscillation amplitude [Figs. 3(c), 6(c), and 6(e)]. The asymmetric atomic distribution also depends on the initial position $\mu \left(z,0\right)$ of each lattice layer [Eq. (5)], indicating that $\mu \left(z,0\right)$ can significantly influence the oscillation of ΔA with frequency ${i\cdot f}_{1}$ under the *i*th order harmonics (*i >* 1). The change in the initial position induced by the lattice distortion was discussed in previous works.^34,35^ However, this was not considered in this work.

The relationship between the frequencies of the diffraction intensity and the coherent phonons now can be clarified. In the breathing or shear modes generated by the homogeneous excitation, the oscillation of the diffraction intensity in $\left(0\bar{2}2\right)$ or (200) with frequency $f_{1}$ is attributed to the fundamental mode, whereas the frequency ${4f}_{1}$ stems from the coupling of the fundamental and third harmonic modes. For the inhomogeneous excitation, additional ${2f}_{1}$ signal is ascribed to the second harmonic mode, whereas the weak ${3f}_{1}$ signal is stemming from the third harmonic mode. It should be mentioned that the finite electron–phonon coupling constant in the actual material would result in a non-zero value of τ. According to the results in Fig. 3(b), the high-frequency signals will be significantly attenuated, and therefore, it is difficult to experimentally observe the ${4f}_{1}$ signal in homogeneous excitation.

It should be noted that the sample is assumed to be absolutely smooth and there are no wrinkles in the film. The LA and TA phonons are simply described by one dimensional wave equation and exist independently in the sample. However, the real material may consist of many wrinkles or micro-patches separated by defects, and each patch may be different in size and orientation, resulting in its own oscillation direction. In addition, TA and LA phonons could be excited simultaneously in the sample, leading to more complicated behavior in oscillation frequency and phase.

## CONCLUSION

In this study, the phase shift and amplitude suppression in oscillation due to the rise time of the stress were analyzed. The results of this work are useful for understanding the generation of coherent phonons under different mechanisms. For example, long electron–phonon coupling time or small thickness can lead to a large relative rise time *τ*/*T*. This significantly weakens the amplitude of thermoelasticity induced coherent phonons, especially the higher harmonics. In addition, we demonstrated the complex behaviors of the diffraction intensity under structural modulation of the coherent LA and TA phonons generated by homogeneous and inhomogeneous distribution. The correspondence between the frequencies of the diffraction intensity oscillation and high harmonics of the strain waves was analyzed by decomposing the diffraction intensity. Thus, the phonon-induced in-phase and out-of-phase intensity oscillation in the Friedel pairs and unequal change were elaborated. These analyses deepened the understanding of the structural behaviors under coherent acoustic phonons and their corresponding influence on electron diffraction, which facilitated further structural modulations by the coherent phonons in the UED device.

## Data Availability

The data that support the findings of this study are available from the corresponding authors upon reasonable request.
